# Testing Nelder-Mead Based Repulsion Algorithms for Multiple Roots of Nonlinear Systems via a Two-Level Factorial Design of Experiments

**DOI:** 10.1371/journal.pone.0121844

**Published:** 2015-04-13

**Authors:** Gisela C. V. Ramadas, Ana Maria A. C. Rocha, Edite M. G. P. Fernandes

**Affiliations:** 1 Department of Mathematics, School of Engineering, Polytechnic of Porto, 4200-072 Porto, Portugal; 2 ALGORITMI Research Centre, University of Minho, Campus de Gualtar 4710-057 Braga, Portugal; University of East Piedmont, ITALY

## Abstract

This paper addresses the challenging task of computing multiple roots of a system of nonlinear equations. A repulsion algorithm that invokes the Nelder-Mead (N-M) local search method and uses a penalty-type merit function based on the error function, known as ‘erf’, is presented. In the N-M algorithm context, different strategies are proposed to enhance the quality of the solutions and improve the overall efficiency. The main goal of this paper is to use a two-level factorial design of experiments to analyze the statistical significance of the observed differences in selected performance criteria produced when testing different strategies in the N-M based repulsion algorithm. The main goal of this paper is to use a two-level factorial design of experiments to analyze the statistical significance of the observed differences in selected performance criteria produced when testing different strategies in the N-M based repulsion algorithm.

## Introduction

In this paper, we aim to investigate the performance of a repulsion algorithm that is based on a penalty function and the Nelder-Mead (N-M) [1] local search procedure to compute multiple roots of a system of nonlinear equations of the form

$$\begin{array}{c} f(x)=0, \end{array}$$ (1)

where *f*(*x*) = (*f*_1_(*x*), *f*_2_(*x*), …, *f*_*n*_(*x*))^*T*^, each *f*_*i*_:Ω ⊂ ℝ^*n*^ → ℝ, *i* = 1, …, *n* is a continuous and possibly nonlinear function in the search space, and Ω is a closed convex set defined as [*l*, *u*] = {*x*:−∞ < *l*_*i*_ ≤ *x*_*i*_ ≤ *u*_*i*_ < ∞, *i* = 1, …, *n*}. Since we do not assume that the functions *f*_*i*_(*x*), *i* = 1, …, *n* are differentiable, neither analytical nor numerical derivatives are used. To compute a solution of a nonlinear system of equations is equivalent to compute a global minimizer of the optimization problem

$$\begin{array}{c} \min_{x\in \Omega \subset {\mathbb{R}}^{n}}\;M\left(x\right)\equiv \sum_{i=1}^{n}f_{i}{\left(x\right)}^{2}, \end{array}$$ (2)

in the sense that they have the same solutions. Thus, a global minimizer and not just a local one, of the function *M*(*x*), known as merit function, in the set Ω, is required. Although finding a single root of a system of nonlinear equations is a trivial task, finding all roots is one of the most demanding problems. Multistart methods are stochastic techniques that have been used to compute multiple solutions to problems [2, 3]. In a multistart strategy, a local search procedure is applied to a set of randomly generated points of the search space to converge sequentially along the iterations to the multiple solutions of the problem, in a single run. However, the same solutions may be located over and over again along the iterations resulting in a very expensive process. Other recent approaches combine metaheuristics with techniques that modify the merit function (2) as solutions are being found [4–8]. Mostly, the techniques rely on the assignment of a penalty term to each previously computed root so that a repulsion area around each previously computed root is created. The repulsion areas force the algorithm to move to other areas of the search space and look for other roots, avoiding repeated convergence to already located solutions. The main idea of this type of methods, designated by repulsion method, is to solve a sequence of global optimization problems aiming to minimize the modified merit function $\bar{M}$ that creates a repulsion area around each computed solution *ξ*_*i*_, for *i* = 1, …, *N*_*r*_, where *N*_*r*_ represents the number of previously computed minimizers, as follows

$$\begin{array}{c} \min_{x\in \Omega \subset {\mathbb{R}}^{n}}\bar{M}\left(x\right)\equiv M\left(x\right)+\sum_{i=1}^{N_{r}}P\left(x;\xi_{i},\cdots \right). \end{array}$$ (3)

The function *P*(*x*;*ξ*_*i*_, ⋯) is the penalty term that depends on *ξ*_*i*_ and one or more parameters [6–8]. The goal of these parameters is to adjust the penalty for already located minimizers. They may be used to reduce the radius of the repulsion area or to highly penalize the proximity to located solutions.

In this study, we further explore this penalty-type approach to create repulsion areas around previously detected roots and propose a repulsion algorithm that is capable of computing multiple roots of a system of nonlinear equations invoking the N-M local procedure with modified merit functions. The herein proposed repulsion algorithm is of a stochastic nature in the sense that a sequence of points are randomly generated inside the search space Ω aiming to increase the exploration ability of the algorithm. The exploitation ability of the algorithm is carried out by implementing the N-M local search starting from each of the generated points. The N-M method is a derivative-free local search procedure that is capable of converging to a minimizer of a merit function, provided that a good initial approximation is given, with a reduced computational effort when compared with most metaheuristics available in the literature.

Design of experiments (DoE) is a powerful statistical tool that is capable of developing an experimentation strategy to learn and identify crucial factors that influence experimental data, using a minimum of resources [9, 10]. Our main challenge here is to analyze the effect of imposing slightly different strategies on the classical N-M algorithm, namely

- randomly generating points for the initial simplex when they fall outside the search space Ω;
- dynamically setting the simplex parameters to generate expansion, contraction and shrinkage vertices;
- generating renewal positions for randomly selected vertices of the simplex to overcome simplex degeneracy;
- generating vertices around the best vertex according to the Lévy distribution to replace the classical shrinkage of the simplex.

We aim to conclude if the observed differences in the performance, measured by different criteria, are considered statistically significant, or are they just explained by normal variation like noise. Usual performance criteria measure robustness and efficiency. For robustness, we consider the percentage of runs that converge to all roots of the system of equations, and for efficiency, we use the number of function evaluations and time required to compute each root.

The paper is organized as follows. First, we address the proposed repulsion algorithm based on an ‘erf’ penalty merit function; second, a summary of the main steps of the classical N-M algorithm, as well as the description of different strategies for testing and performance assessment are presented. Afterwards, a two-level factorial DoE is introduced and the corresponding statistical analysis is carried out. Finally, a performance comparison is reported and the conclusions are presented.

## Repulsion algorithm

This section aims to briefly describe a penalty-type approach to create repulsion areas around previously computed roots of a system of nonlinear equations, so that convergence to already located solutions is avoided. Algorithms based on repulsion merit functions have been used for locating multiple roots of systems of equations [6, 8]. The merits of a repulsion algorithm are that multiple roots can be located with a reduced computational effort in a single run of the algorithm. The pseudo code of the repulsion algorithm is presented in Algorithm 1 in Table 1.

**Table 1:** Algorithm 1.

**Require**: *k_R_*_max_ > 0, ϵ > 0;	
1:	Set Ξ = ∅, *k_R_* = 0, *k_uns_* = 0, *N_r_* = 0;
2:	Randomly generate *y* in Ω;
3:	Given *y*, compute *ξ*_1_ = arg min_*x*∈Ω_ *M*(*x*);
4:	**if** *ξ*_1_ is a root **then**
5:	Set *N_r_* = 1, *r*_1_ = 1, Ξ = Ξ ∪ *ξ*_1_;
6:	**else**
7:	Set *k*_uns_ = *k*_uns_ + 1;
8:	**end if**
9:	**while** stopping conditions are not met **do**
10:	Randomly generate *y* in Ω;
11:	Given *y*, compute $\xi =\text{arg}\;{\text{min}}_{x\in \Omega }\;\bar{M}\;(x)$
12:	**if** *ξ* is a root **then**
13:	**if** *ξ* ∉ Ξ **then**
14:	Set *N_r_* = *N_r_* + 1, *ξ_N_r__* = *ξ*, *r_N_r__* = 1, Ξ = Ξ ∪ *ξ_N_r__*;
15:	**else**
16:	Set *r_l_* = *r_l_* + 1 (*ξ_l_* ∈ Ξ);
17:	**end if**
18:	*k*_uns_ = 0;
19:	**else**
20:	Set *k*_uns_ = *k*_uns_ + 1;
21:	**end if**
22:	Set *k_R_* = *k_R_* + 1;
23:	**end while**

Repulsion Algorithm

The algorithm solves a sequence of global optimization problems by invoking a local search procedure that is capable of locating a local minimizer of a merit function. This function is modified as the roots are located. See steps 1 and 1 of Algorithm 1 in Table 1. The first call to the local search considers the original merit function (2). Thereafter the merit function is modified to avoid locating previously computed minimizers.

In the algorithm, ‘*k*_*R*_’ is the iteration counter, ‘Ξ’ is the set where the roots are saved, ‘*N*_*r*_’ contains the number of located roots so far, ‘*r*_1_, …, *r*_*N*_*r*__’ are variables that contain the number of times each root is relocated and ‘*k*_uns_’ represents the number of consecutive unsuccessful iterations, i.e., consecutive iterations that are not able to find any new root. To identify a root of the system the algorithm requires that its merit function value is under 10^−10^. A located root *ξ* is considered to be different from the previously computed roots *ξ*_*i*_, *i* = 1, …, *N*_*r*_ (saved in Ξ) if ‖*ξ*−*ξ*_*i*_‖ > *ε*, for all *i* = 1, …, *N*_*r*_, where *ε* is a small positive error tolerance.

The repulsion algorithm terminates when a maximum number of iterations, *k*_*R*max_, is exceeded or when a pre-specified number of consecutive iterations (15, in this algorithm) are not able to locate any new root. Thus, if

$$\begin{array}{c} k_{R}>k_{R\max}\;\;\text{or}\;\;k_{\text{uns}}>15 \end{array}$$

the algorithm stops.

The algorithm explores the search space by randomly generating points in Ω in a sequential manner (see steps 1 and 1 of the algorithm). Starting from the sampled point *y*, the algorithm exploits the region in order to locate a minimizer of the merit/modified merit function by invoking a local search procedure (see steps 1 and 1 of Algorithm 1 in Table 1). Since the goal is to produce a derivative-free effective algorithm for locating multiple roots, our proposal for computing a minimizer of the merit function, given a sampled point *y*, is the N-M local procedure. This is the subject of the next section.

**Fig 1:**
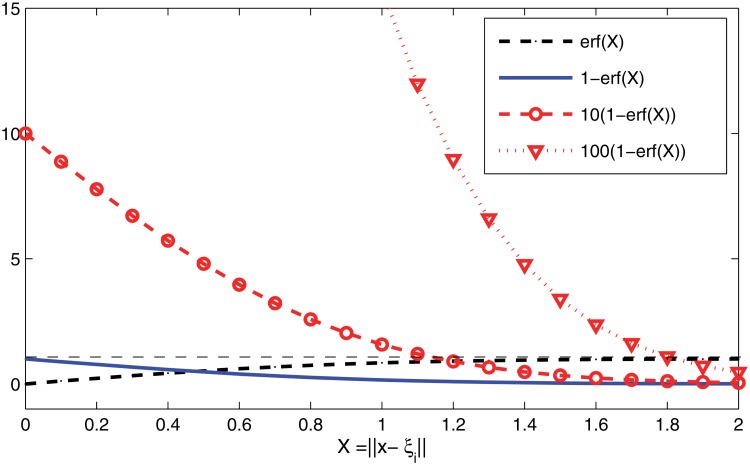
The ‘erf’ penalty for different *δ*.

The herein implemented penalty-type merit function is based on the error function, known as ‘erf’, which is a mathematical function defined by the integral

$$\begin{array}{c} \mathrm{erf}\left(x\right)=\frac{2}{\sqrt{\pi }}\int_{0}^{x}e^{-t^{2}}\;dt, \end{array}$$

and satisfies the following properties erf(0) = 0, erf(−∞) = −1, erf(+∞) = 1 and erf(−*x*) = −erf(*x*). In a penalty function context aiming to prevent convergence to a located root *ξ*_*i*_, thus defining a repulsion area around it, the newly developed ‘erf’ penalty term is:

$$\begin{array}{c} P\left(x;\xi_{i},\delta ,\rho \right)=\left\{\begin{array}{c} \delta \left(1-\mathrm{erf}(∥x-\xi_{i}∥)\right),\\ \;\;\text{if}\;∥x-\xi_{i}∥\leq \rho \\ 0,\;\text{otherwise} \end{array}\right. \end{array}$$ (4)

which depends on the parameter *δ* > 0 to scale the penalty for approaching the already computed solution *ξ*_*i*_ and on the parameter *ρ* to adjust the radius of the repulsion area. To better understand the effect of the ‘erf’ penalty around 0, we plot in Fig 1 the function (4) for different values of the parameter *δ* (1, 10 and 100).

We note that the penalty term tends to *δ* when *x* approaches a root *ξ*_*i*_, meaning that *δ* should be made large enough so that *ξ*_*i*_ is no longer a minimizer of the modified penalty merit function. Further, as *x* moves away from *ξ*_*i*_, the penalty *P*(*x*;*ξ*_*i*_, *δ*, *ρ*) → 0 meaning that the modified merit function $\bar{M}(x)=M(x)+P(x;\xi_{i},\delta ,\rho )$ is not affected when *x* is far from a previously located root.

## N-M Algorithm Variants

The N-M algorithm, also known as simplex search algorithm, originally published in 1965 [1], is probably the best known algorithm for multidimensional derivative-free optimization, and is an improvement over the Spendley’s et al. method [11].

In ℝ^*n*^, the N-M algorithm uses a set of *n*+1 vertices, by now denoted by *x* ^1^, *x* ^2^, …, *x* ^*n*^, *x* ^*n*+1^, to define a simplex, which is an *n*-dimensional polytope—the convex hull of its *n*+1 vertices. The algorithm moves at least one vertex per iteration defining a new simplex for the next iteration. The main idea is to maintain at each iteration a nondegenerate simplex, i.e., a geometric figure in ℝ^*n*^ of nonzero volume. In [11], either a reflection or a shrink step is performed so that the shape of the simplex does not change along the iterative process. In general, a reflection step is performed when the current simplex is far from the solution, while a shrink step is performed when the simplex is close to the solution. In terms of computational effort, when a reflection step is performed only one or two function evaluations are required, while when a shrink step is performed, *n* function evaluations are required.

The initial simplex could be computed by performing small perturbations around an initial guess point. Thus, given *x* ^1^, an approximation to the minimizer of *M* (or, it may be a randomly generated point in Ω), the other *n* vertices are usually generated along the unit coordinate vectors *e*_*j*_ ∈ ℝ^*n*^ with a fixed step size *ɛ*_*e*_ ∈ (0,1] (at an equal distance relative to *x* ^1^) as follows:

$$\begin{array}{c} x^{j}=x^{1}+\varepsilon_{e}e_{j-1}\;,\;\;\;j=2,\ldots ,n+1. \end{array}$$

It has been observed that using short edges in the initial simplex is a good strategy for small dimensional problems [12]. Although this is the most popular rule to generate the initial simplex, when the problem contains bound constraints, some (or all) components of the other vertices *x* ^*j*^ (*j* = 2, …, *n*+1) may fall outside the search space Ω, even if *x* ^1^ is inside. Two possible ways of restoring feasibility are: (i) project the component of the point onto the boundary of Ω, as shown in Algorithm 2 in Table 2; or (ii) randomly generate the component of the point inside Ω, as described in Algorithm 3 in Table 3, where *τ* is a uniformly distributed random number in [0, 1] (*τ* ∼ *U*[0, 1]).

**Table 2:** Algorithm 2.

**Require:** *n* *ϵ_e_* > 0, *x* ^1^ ∈ [*l*, *u*]	
1:	Set *e_j_* to *j*th. column of the identify matrix, for *j* = 1, …, *n*
2:	**for all** *j* = 2, …, *n* + 1 **do**
3:	compute *x^j^* = *x* ^1^ + *ϵ_e_e*_*j*–1_
4:	**for all** *i* = 1, …, *n* **do**
5:	**if** $x_{i}^{j}<l_{i}$ **then**
6:	Set $x_{i}^{j}=l_{i}$
7:	**else if** $x_{i}^{j}>u_{i}$ **then**
8:	Set $x_{i}^{j}=u_{i}$
9:	**end if**
10:	**end for**
11:	**end for**

Initialization with projection

**Table 3:** Algorithm 3.

**Require:** *n*, *ϵ_e_* > 0, *x^1^* ∈ [*l*, *u*]	
1:	Set *e_j_* to *j*th. column of the identify matrix, for *j* = 1, …, *n*
2:	**for all** *j* = 2, …, *n* + 1 **do**
3:	compute *x^j^* = *x^1^* + *ϵ_e_e*_*j*–1_
4:	**for all** *i* = 1, …, *n* **do**
5:	**if** $x_{i}^{j}<l_{i}$ or $x_{i}^{j}>u_{i}$ **then**
6:	Compute $x_{i}^{j}=l_{i}+\tau (u_{i}-l_{i})$
7:	**end if**
8:	**end for**
9:	**end for**

Initialization with random components

To define a set of candidate points to be a vertex of the simplex of the next iteration, the simplex is ordered so that *M*(*z* ^1^) ≤ *M*(*z* ^2^) ≤ ⋯*M*(*z* ^*n*^) ≤ *M*(*z* ^*n*+1^), where *z* ^1^ is termed the best vertex, *z* ^*n*+1^ the worst vertex and *z* ^*n*^ the second-worst (or next-worst) of the simplex. Note that simplex ordering relabels the vertices according to merit function value and are now represented by *z* ^1^, *z* ^2^, …, *z* ^*n*^, *z* ^*n*+1^. Let the centroid of the *n* best vertices be defined by

$$\begin{array}{c} \bar{x}=\frac{1}{n}\sum_{i=1}^{n}z^{i} \end{array}$$

then the new candidate points to a vertex are defined along the line defined by $\bar{x}$ and *z* ^*n*+1^. They are constructed componentwise as follows

$$\begin{array}{c} \left.\begin{array}{c} x_{r,j}\\ x_{e,j}\\ x_{oc,j}\\ x_{ic,j} \end{array}\right\}={\bar{x}}_{j}+\gamma \left({\bar{x}}_{j}-z_{j}^{n+1}\right) \end{array}$$ (5)

for *j* = 1, …, *n*, where *x*_*r*_, *x*_*e*_, *x*_*oc*_ and *x*_*ic*_, usually referred to as reflection vertex, expansion vertex, outer contraction vertex and inner contraction vertex, are obtained setting *γ* to *γ*_*r*_, *γ*_*e*_, *γ*_*oc*_ and *γ*_*ic*_ respectively. We remark that the algorithm checks if the component of the vector in (5) stays inside Ω; otherwise, a projection onto the boundary is carried out. The rules that are used to accept one of the above referred candidate points are directed related with their objective function values relative to the function values of the best vertex, second-worst vertex and worst vertex, are shown in Algorithm 4 in Table 4, and are according to [13]. There might be some differences in the literature, for instances in [1]. When one of these vertices is accepted, the worst vertex is discarded and the new point takes the position of a vertex of the simplex for the next iteration.

**Table 4:** Algorithm 4.

**Require:** *n*, *k*_max_, *ϵ*, *x* ^1^ ∈ Ω	
1:	Set *k* = 0
2:	Generate the remaining *n* vertices *x* ^2^, …, *x* ^*n*+1^ using Algorithm 2 in Table 2 or Algorithm 3 in Table 3
3:	Order the simplex: *M*(*z* ^1^) ≤ *M*(*z* ^2^) ≤ ⋯ *M*(*z^n^*) ≤ *M*(*z* ^*n*+1^)
4:	Define the simplex parameters by (7) or (8)
5:	**while** *M*(*z* ^1^) > 10^-10^ and *k* ≤ *k*_max_ **do**
6:	Compute *V* and det(*V*) (see (9))
7:	**if** simplex has collapsed **then**
8:	‘break’ or generate vertices using (11) with probability 0.75
9:	Order the simplex: *M*(*z* ^1^) ≤ *M*(*z* ^2^) ≤ ⋯ *M*(*z^n^*) ≤ *M*(*z* ^*n*+1^)
10:	**end if**
11:	Compute *x_r_* using (5) and evaluate *M*(*x_r_*)
12:	**if** *M*(*x_r_*) < *M*(*z* ^1^) **then**
13:	Compute *x_e_* using (5) and evaluate *M*(*x_e_*)
14:	**if** *M*(*x_e_*) < *M*(*x_r_*) **then**
15:	Accept *x_e_*
16:	**else**
17:	Accept *x_r_*
18:	**end if**
19:	**else if** *M*(*z* ^1^) ≤ *M*(*x_r_*) < *M*(*z^n^*) **then**
20:	Accept *x_r_*
21:	**else if** *M*(*z^n^*) ≤ *M*(*x_r_*) < *M*(*z* ^*n*+1^) **then**
22:	Compute *x_oc_* using (5) and evaluate *M*(*x_oc_*)
23:	**if** *M*(*x_oc_*) ≤ *M*(*x_r_*) **then**
24:	Accept *x_oc_*
25:	**else**
26:	*x* ^1^ ← *z* ^1^ and compute *x^i^*, *i* = 2, 3, …, *n* + 1 using (6) or (12)
27:	**end if**
28:	**else**
29:	Compute *x_ic_* using (5) and evaluate *M*(*x_ic_*)
30:	**if** *M*(*x_ic_*) ≤ *M*(*z* ^*n*+1^) **then**
31:	Accept *x_ic_*
32:	**else**
33:	*x* ^1^ ← *z* ^1^ and compute *x^i^*, *i* = 2, 3, …, *n* + 1 using (6) or (12)
34:	**end if**
35:	**end if**
36:	Order the simplex: *M*(*z* ^1^) ≤ *M*(*z* ^2^) ≤ ⋯ *M*(*z^n^*) ≤ *M*(*z* ^*n*+1^)
37:	Set *k* = *k* + 1
38:	**end while**

N-M Algorithm

In classical versions of the N-M method [1, 13–17], when none of these vertices is accepted, according to the rules described in Algorithm 4 in Table 4, the simplex is shrunk towards the best vertex by using

$$\begin{array}{c} x_{j}^{i}=z_{j}^{1}+\gamma_{s}\left(z_{j}^{i}-z_{j}^{1}\right) \end{array}$$ (6)

for *i* = 2, …, *n*+1 and *j* = 1, …, *n*. According to [1], the simplex *γ* parameters that give the step size to generate the points in (5) and (6) must satisfy 0 < *γ*_*r*_ < *γ*_*e*_, *γ*_*e*_ > 1, −1 < *γ*_*ic*_ < 0 < *γ*_*oc*_ < 1 and 0 < *γ*_*s*_ < 1. The most commonly used values for the parameters are:

$$\begin{array}{c} \gamma_{r}=1,\;\gamma_{e}=2,\;\gamma_{oc}=0.5,\;\gamma_{ic}=-\gamma_{oc},\;\gamma_{s}=0.5\;. \end{array}$$ (7)

The examples reported in [15] showing that N-M may converge to a non-critical point of a strictly convex objective function are well-known in the community. N-M is one of the most popular direct search method and has shown to behave rather well when solving small dimensional problems. Although it has been used in many scientific and engineering applications, there are just a few works on the convergence properties of the algorithm [12, 13, 15, 16]. The performance of N-M gets worse as *n* increases. The authors in [12] prove that the efficiency of the expansion and contraction steps diminishes as *n* increases when N-M relies on the standard parameter values (see in (7)) to solve uniformly convex objective functions. Thus, values for the *γ* parameters for expansion, contraction and shrinking, dependent on *n*, are proposed in [12] to improve convergence. The golden ratio represented by the Greek letter Φ has been used in many practical situation to bring balance and harmony in life. It is a real number and is the unique positive solution of the quadratic equation Φ^2^−Φ−1 = 0. Here, we propose to define the N-M *γ* parameters using the golden value $\Phi =\frac{\sqrt{5}+1}{2}$ and *n* in order to have balanced expanded and reduced simplices, as follows:

$$\begin{array}{ccc} \gamma_{r} & = & 1,\;\gamma_{e}=\Phi +\frac{1}{n},\;\gamma_{oc}=\Phi -1-\frac{1}{n},\\ \gamma_{ic} & = & -\gamma_{oc},\;\gamma_{s}=\Phi -1-\frac{1}{n}. \end{array}$$ (8)

Note that we propose to use *γ*_*r*_ = 1 in order to keep the isometric reflection since this is crucial for good performance [12, 16]. We also remark that expansion, contraction and shrinkage vertices are obtained from (5) and (6), using (8) to define the associated parameters dependent on the problem’s dimension. The goal is to reduce the simplex in a smoother way.

The major difficulty with the N-M method is that a sequence of simplices can come arbitrarily close to degeneracy. Let *V* be the *n*×*n* matrix with *n*-vector columns *v* ^1^, *v* ^2^, …, *v* ^*n*^ where

$$\begin{array}{c} V\equiv \left(v^{1}\;\cdots \;v^{n}\right)=\left(\begin{array}{ccc} z_{1}^{2}-z_{1}^{1} & \cdots & z_{1}^{n+1}-z_{1}^{1}\\ & \ddots & \\ z_{n}^{2}-z_{n}^{1} & \cdots & z_{n}^{n+1}-z_{n}^{1} \end{array}\right) \end{array}$$ (9)

and det(*V*) denote the determinant of *V*. To keep the interior angles of the simplex bounded away from 0, the following condition has to be maintained along the process

$$\begin{array}{c} \left|\det(V)\right|\geq \epsilon_{v}\prod_{j=1}^{n}∥v^{j}∥ \end{array}$$ (10)

where *ε*_*v*_ is a small positive constant. When (10) fails to be satisfied, the simplex has collapsed and needs to be reshaped. The simplest option is to ‘break’ the iterative process and return to the main repulsion algorithm with no solution. Here, we are interested in testing a renewal strategy that aims to keep the best vertex and has a 75% chance of changing the remaining *n* vertices (*i* = 2, …, *n* + 1), componentwise, as follows:

$$\begin{array}{c} x_{j}^{i}=z_{j}^{1}\pm \tau \;\Phi \left(u_{j}-l_{j}\right) \end{array}$$ (11)

using again the golden ratio to balance the step size of the move around *z* ^1^. If the generated components fall outside Ω, a projection onto the boundary is carried out.

When none of the *x*_*r*_, *x*_*e*_, *x*_*oc*_ and *x*_*ic*_ vertices is accepted, a procedure based on the randomly generation of points according to a Lévy distribution is also proposed. This is a stable and heavy-tailed distribution that is able to provide occasionally long movements. Similarly to the classical shrinkage procedure (6), the best vertex is maintained, and the other *n* vertices are generated around *z* ^1^:

$$\begin{array}{c} x_{j}^{i}=z_{j}^{1}+L\left(\alpha \right)\sigma_{j}^{i}\;, \end{array}$$ (12)

for *i* = 2, …, *n*+1 and *j* = 1, …, *n*, where *L*(*α*) is a random number from the standard Lévy distribution, with location parameter equals to 0, a unit scale parameter, and the shape parameter *α* = 0.5. Again, if the components fall outside Ω, a projection onto the boundary is carried out. The vector *σ* ^*i*^ in (12) represents the variation around *z* ^1^

$$\begin{array}{c} \sigma^{i}={\left(|z_{1}^{i}-z_{1}^{1}\left|,\ldots ,\right|z_{n}^{i}-z_{n}^{1}|\right)}^{T}. \end{array}$$

## Two-Level Factorial DoE

In this section, we aim to use a two-level factorial DoE to analyze the effect of imposing the above referred strategies on the N-M algorithm, as well as to know if the observed differences in selected performance criteria are considered statistically significant or are they just due to random variations from normal distribution. Some notation and terminology related to factorial DoE will be introduced. In the previous section, we considered different strategies that can be implemented and manipulated within the N-M algorithm, in order to improve its performance. We aim to analyze the effect of those strategies—hereafter denoted by ‘factor levels’ or ‘treatments’—on the performance of the algorithm. For the design of an experiment, the following main steps must be carried out [9, 10]:

- formulation of the statistical hypotheses;
- definition of the factors and their levels (independent variables) and the measurements (response or dependent variables) to be recorded;
- specification of the number of experimental units;
- specification of the randomization process to assign the experimental units to the treatments;
- determination of the statistical analysis.

The simplest DoE involves randomly assigning experimental units to a factor with two or more levels. However, when more than one factor require to be analyzed, a factorial DoE is preferable. The simplest one involves two factors, each at two levels, denoted by two-by-two design. To analyze the two factor effects—named ‘A’ and ‘B’ –, a set of four experimental conditions should be analyzed. Let us denote the two levels of each factor by ‘low’ (or ‘-1’) and ‘high’ (or ‘+1’). Then, the first experiment combines the low level of A with the low level of B, the second combines the high level of A with the low level of B, the third combines the low level of A with the high level of B, and the fourth combines the high levels of both A and B. Although factorial experiments can involve factors with different number of levels, we are interested in a design where all factors have two levels. We plan to use a factorial DoE with four factors, each at two levels, and overall 2^4^ experimental conditions are to be conducted. In the statistical field, each experimental condition represents a different ‘treatment’ protocol.

We have chosen sixteen problems with different characteristics, dimensions and different number of roots to test the 2^4^ ‘treatments’. The problems and the number of roots are listed in Table 5 and are available in the literature [3, 5, 6, 8, 18, 19]. Due to the stochastic nature of the repulsion algorithm, from which N-M is invoked, we run each ‘treatment’ 30 times with each problem. The results are averaged over the 30 runs for each problem.

**Table 5:** Problems set.

NonD2	$f_{1}=x_{1}^{2}-x_{2}^{2}$
	*f*_2_ = 1 − ∣*x*_1_ − *x*_2_∣
	2 roots in [−3, 3]^2^
Trans	$f_{1}=x_{1}^{2}-x_{2}-2$
	*f*_2_ = *x*_1_ + sin(*πx*_2_/2)
	3 roots in [−3, 3]^2^
Himmelblau	$f_{1}=4x_{1}^{3}+4x_{1}x_{2}+2x_{2}^{2}-42x_{1}-14$
	$f_{2}=4x_{2}^{3}+4x_{1}x_{2}+2x_{1}^{2}-26x_{2}-22$
	9 roots in [−5, 5]^2^
Geometry	*f*_1_ = *x*_1_ *x*_2_ + (*x*_1_ − 2*x*_3_)(*x*_2_ − 2*x*_3_) − 165
	$f_{2}=(x_{1}x_{2}^{3})/12-(x_{1}-2x_{3}){\left(x_{2}-2x_{3}\right)}^{3}/12-9369$
	*f*_3_ = (2(*x*_2_ − *x*_3_)^2^(*x*_1_ − *x*_3_)^2^ *x*_3_)/(*x*_1_ + *x*_2_ − 2*x*_3_) − 6835
	2 roots in [0, 50]^3^
Floudas	*f*_1_ = (0.25/*π*)*x*_2_ + 0.5*x*_1_ − 0.5 sin(*x*_1_ *x*_2_)
	$f_{2}=(e/\pi )x_{2}-2ex_{1}+(1-0.25/\pi )(e^{2x_{1}}-e)$
	2 roots in [0.25, 1] × [1.5, 2*π*]
Merlet	*f*_1_ = − sin(*x*_1_) cos(*x*_2_) − 2 cos(*x*_1_) sin(*x*_2_)
	*f*_2_ = − cos(*x*_1_) sin(*x*_2_) − 2 sin(*x*_1_) cos(*x*_2_)
	13 roots in [0, 2*π*]^2^
Reactor	*f*_1_ = (1 − *R*) (*D*/(10(1 + *B*_1_)) − *x*_1_) *e* ^10*x*_1_/(1 + 10*x*_1_/*γ*)^ − *x*_1_
	*f*_2_ = (1 − *R*) (*D*/10 − *B*_1_ *x*_1_ − (1 + *B*_2_)*x*_2_))*e* ^10*x*_2_/(1 + 10*x*_2_/*γ*)^ + *x*_1_ − (1 + *B*_2_)*x*_2_ with *D* = 22, *B*_1_ = *B*_2_ = 2, *R* = 0.960 and *γ* = 1000
	7 roots in [0, 1]^2^
P1syst	*f*_1_ = *x*_1_ + *x*_2_ − 3
	$f_{2}=x_{1}^{2}+x_{2}^{2}-9$
	2 roots in [−3, 3]^2^
Papersys	*f*_1_ = *x*_1_ − sin(2*x*_1_ + 3*x*_2_) − cos(3*x*_1_ − 5*x*_2_)
	*f*_2_ = *x*_2_ − sin(*x*_1_ − 2*x*_2_) + cos(*x*_1_ + 3*x*_2_)
	3 roots in [−3, 3]^2^
Casestudy5	$f_{1}=e^{x_{1}^{2}}-8x_{1}\sin(x_{2})$
	*f*_2_ = *x*_1_ + *x*_2_ − 1
	*f*_3_ = (*x*_3_ − 1)^3^
	2 roots in [0, 1]^3^
Casestudy7	$f_{1}=x_{1}^{3}-3x_{1}x_{2}^{2}-1$
	$f_{2}=3x_{1}^{2}x_{2}-x_{2}^{3}+1$
	3 roots in [−1, 2]^2^
Parsopoulos	*f*_1_ = cos(*x*_1_)
	*f*_2_ = sin(*x*_2_)
	12 roots in [−5, 5]^2^
Robot	*f*_1_ = −0.1238*x*_1_ + *x*_7_ − 0.001637*x*_2_ − 0.9338*x*_4_ + 0.004731*x*_1_ *x*_3_ − 0.3578*x*_2_ *x*_3_ − 0.3571
	*f*_2_ = 0.2638*x*_1_ − *x*_7_ − 0.07745*x*_2_ − 0.6734*x*_4_ + 0.2238*x*_1_ *x*_3_ + 0.7623*x*_2_ *x*_3_ − 0.6022
	*f*_3_ = 0.3578*x*_1_ + 0.004731*x*_2_ + *x*_6_ *x*_8_ *f*_4_ = −0.7623*x*_1_ + 0.2238*x*_2_ + 0.3461
	$f_{5}=x_{1}^{2}+x_{2}^{2}-1$ $f_{6}=x_{3}^{2}+x_{4}^{2}-1$
	$f_{7}=x_{5}^{2}+x_{6}^{2}-1$ $f_{8}=x_{7}^{2}+x_{8}^{2}-1$
	16 roots in [−1, 1]^8^
NonDif	$f_{1}=3-x_{1}x_{3}^{2}$
	*f*_2_ = *x*_3_ sin(*π*/*x*_2_) − *x*_3_ − *x*_4_
	$f_{3}=-x_{2}x_{3}e^{1-x_{1}x_{3}}+0.2707$
	$f_{4}=2x_{1}^{2}x_{3}-x_{2}^{4}x_{3}-x_{2}$
	1 root in [0, 4]^4^
Ex5_6	$f_{1}=x_{1}^{2}+x_{2}^{2}+x_{3}^{2}-9$
	*f*_2_ = *x*_1_ *x*_2_ *x*_3_ − 1
	$f_{3}=x_{1}+x_{2}-x_{3}^{2}$
	2 roots in [0, 3]^3^
Manipulator	$f=3.9852-10.039x^{2}+7.2338x^{4}-1.17775x^{6}+(-8.8575x+20.091x^{3}-11.177x^{5})\sqrt{1-x^{2}}$
	6 roots in [−1, 1]

Problems set

Three response variables are considered to analyze the performance of the algorithm variants:

- the proportion of successful runs, ranging from 0 to 1, and hereafter denoted by *Y*_*p*_;
- the number of function evaluations to locate each root, ranging from 0 to 1500, denoted by *Y*_*e*_;
- the time (in seconds) to locate each root, ranging from 0 to 1, denoted by *Y*_*t*_.

To compute *Y*_*p*_, a run is considered to be successful if all the roots are located at that run. To take into consideration the diversity of problems, the values of the above response variables for the statistical analysis are averaged over the sixteen problems. At the end, for each ‘treatment’ an average value of a total of 480 results is considered in this experiment.

### Parameters setting

All the experiments were carried out in a PC Intel Core 2 Duo Processor E7500 with 2.9GHz and 4Gb of memory. The algorithms were coded in Matlab Version 8.0.0.783 (R2012b). The parameters have been set after an empirical study. For the Algorithm 1, we consider: *k*_*R*max_ = 50, *ε* = 0.005, *δ* = 100 and *ρ* = min{*ρ*_*ε*_, min_*i*_‖*x*−*ξ*_*i*_‖}, where *ρ*_*ε*_ is set to 1 for the Geometry problem, to 0.1 for Trans, Parsopoulos and NonDif problems, to 0.001 for NonD2, Floudas and Robot and to 0.01 for the remaining problems. In the N-M algorithm, we use *ɛ*_*e*_ = 0.1, *ε*_*v*_ = 10^−8^ and *k*_max_ depends on the problem’s dimension and is set to 100*n* ^2^.

### Factor and interaction effects

The four factors, denoted for simplicity by A, B, C and D, that are manipulated in the N-M algorithm in order to analyze the effect on the performance of the repulsion algorithm are the following.

**Factor A** is directly related with the initialization of the simplex. This is a factor of categorical type. Either Algorithm 2 in Table 2 or Algorithm 3 in Table 3 is analyzed. The low level (-1) of factor A represents the Algorithm 2 and the high level (+1) represents Algorithm 3.

**Factor B** is related with the values set to the simplex *γ* parameters and is a numerical type factor. The standard values in (7) define the low level and the values herein proposed in (8) define the high level of the factor.

**Factor C** is related with the strategy to be used to overcome the simplex degeneracy. This is also a categorical type factor. The low level represents the ‘break’ strategy and the high level represents the generation of new vertices using (11) with probability 0.75.

**Factor D** has to do with the adopted strategy when none of the reflection, expansion and contraction vertices is accepted. The low level represents the usual shrinkage strategy, as shown in (6), while the high level aims to represent the generation of new vertices by the Lévy distribution using (12).

The results obtained from the combination of the four factors, each at two levels, are shown in Table 6. The layout is a standard one, starting with all factors at low levels and ending with all factors at high levels. Interactions occur when the effect of one factor depends on the level of the other. They cannot be detected by an one-factor-at-a-time experimentation. This factorial design also allows the estimation of six 2-factor interactions (AB, AC, AD, BC, BD, CD), four 3-factor interactions (ABC, ABD, ACD, BCD) and one 4-factor interaction (ABCD), a total of 15 effects. (The most we can estimate from a ‘four factors at two levels’ DoE, because 1 degree of freedom is used to estimate the overall mean.) The ± signs for interaction effects are computed by multiplying the signs of the factors involved in the interaction. After conducting the experiments, the observed averaged values of the response variables are reported in the last three columns of Table 6.

**Table 6:** Layout for the 2^4^ factorial design.

‘treatment’	factor effects				interaction effects											response		
	A	B	C	D	AB	AC	AD	BC	BD	CD	ABC	ABD	ACD	BCD	ABCD	*Y*_*p*_	*Y*_*e*_	*Y*_*t*_
1	-1	-1	-1	-1	1	1	1	1	1	1	-1	-1	-1	-1	1	0.55	209.2	0.028
2	1	-1	-1	-1	-1	-1	-1	1	1	1	1	1	1	-1	-1	0.57	207.9	0.028
3	-1	1	-1	-1	-1	1	1	-1	-1	1	1	1	-1	1	-1	0.56	286.6	0.036
4	1	1	-1	-1	1	-1	-1	-1	-1	1	-1	-1	1	1	1	0.58	283.6	0.035
5	-1	-1	1	-1	1	-1	1	-1	1	-1	1	-1	1	1	-1	0.69	292.3	0.037
6	1	-1	1	-1	-1	1	-1	-1	1	-1	-1	1	-1	1	1	0.68	305.4	0.037
7	-1	1	1	-1	-1	-1	1	1	-1	-1	-1	1	1	-1	1	0.72	391.7	0.047
8	1	1	1	-1	1	1	-1	1	-1	-1	1	-1	-1	-1	-1	0.77	400.4	0.048
9	-1	-1	-1	1	1	1	-1	1	-1	-1	-1	1	1	1	-1	0.57	201.0	0.027
10	1	-1	-1	1	-1	-1	1	1	-1	-1	1	-1	-1	1	1	0.56	203.1	0.027
11	-1	1	-1	1	-1	1	-1	-1	1	-1	1	-1	1	-1	1	0.58	302.7	0.036
12	1	1	-1	1	1	-1	1	-1	1	-1	-1	1	-1	-1	-1	0.56	293.1	0.036
13	-1	-1	1	1	1	-1	-1	-1	-1	1	1	1	-1	-1	1	0.72	345.7	0.042
14	1	-1	1	1	-1	1	1	-1	-1	1	-1	-1	1	-1	-1	0.73	334.3	0.041
15	-1	1	1	1	-1	-1	-1	1	1	1	-1	-1	-1	1	-1	0.74	409.6	0.050
16	1	1	1	1	1	1	1	1	1	1	1	1	1	1	1	0.74	389.9	0.046
*E*_*e*_ on *Y*_*p*_	0.010	0.024	0.158	0.010	0.005	0.005	-0.012	0.013	-0.014	0.008	0.006	-0.008	0.007	-0.011	-0.006			
*E*_*e*_ on *Y*_*e*_	-2.628	82.315	110.260	12.767	-3.237	0.297	-7.003	-3.882	-4.526	9.637	0.077	-1.731	-6.234	-14.185	0.783			
*E*_*e*_ on *Y*_*t*_	-0.001	0.0083	0.012	0.001	-0.0004	-0.0002	-0.001	0.0001	-0.0003	0.002	-0.0002	-0.001	-0.001	-0.001	-0.0003			

Layout for the 2^4^ factorial design

Replication is an important principle in experimentation and is concerned with using more than one experimental unit under the same conditions. When replication is used, error effects can be estimated. Although we have multiple response observations taken at the same factor levels, they cannot be considered replications since these would have been recorded in a random order. Furthermore, the stochastic nature of the repulsion algorithm dictates that the experimental conditions would not be at all the same over the replications.

Let *Y* ^*i*^ represent the average value of all the obtained results when treatment *i* is conducted ($i=1,\ldots ,2^{N_{f}}$). To estimate the effect of each factor or interaction on each response variable (*Y*_*p*_, *Y*_*e*_ and *Y*_*t*_), we analyze the difference or contrast between the average of the highs and the average of the lows. Mathematically, the effect can be estimated using

$$\begin{array}{c} E_{e}=\frac{1}{2^{N_{f}-1}}\left(\pm Y^{1}\pm Y^{2}\pm \cdots \pm Y^{2^{N_{f}}}\right) \end{array}$$ (13)

where *N*_*f*_ represents the number of factors in this two-level analysis, each ± sign corresponds to the sign of the respective factor/interaction column in Table 6 and the $Y^{1},\ldots ,Y^{2^{N_{f}}}$ are the averaged values obtained for each associated response variable, during the $2^{N_{f}}$ conducted ‘treatments’.

The estimated effects computed by (13) are shown in the last three rows of Table 6. It looks like factor C has a high impact on the response variable *Y*_*p*_, with *E*_*e*_ = 0.158, (which aims to measure algorithm robustness) and factor B has a moderate effect on *Y*_*p*_ (*E*_*e*_ = 0.024). Factors A and D have very little effect on *Y*_*p*_ (with *E*_*e*_ = 0.010 for both factors). Further, factors B and C have a much larger effect on the variable *Y*_*e*_, with *E*_*e*_ equal to 82.315 and 110.260 respectively, (which gives algorithm efficiency) than factors A and D (*E*_*e*_ = −2.628 and *E*_*e*_ = 12.767). Finally, factor C greatly affects variable *Y*_*t*_, with *E*_*e*_ = 0.012, (also measuring the efficiency), when compared with factor B that affects moderately the variable *Y*_*t*_ (*E*_*e*_ = 0.0083), while factors A (*E*_*e*_ = −0.001) and D (*E*_*e*_ = 0.001) have little effect on time.

A simple analysis allows us to conclude that factor C produces the highest effect on the response variables. It is noticed that the results obtained when factor C is at the low level are consistently lower than those obtained when C is at high level. This means that when the simplex collapses, the strategy that generates new vertices using (11) with probability 0.75, when compared with the strategy that ‘breaks’ the cycle: (i) has a positive impact on *Y*_*p*_ (with *E*_*e*_ = 0.158), which is good since the percentage of successful runs increased; (ii) has a positive impact on *Y*_*e*_ (with *E*_*e*_ = 110.260), increasing the number of function evaluations which means that the strategy is computationally more demanding; and (iii) also has a positive impact on *Y*_*t*_ (with *E*_*e*_ = 0.012), meaning that the time has increased worsening the efficiency.

A similar reasoning can be applied to the factor B related with the choice of *γ* parameters reported in (8), when compared with (7), although with a more moderate impact.

As far as the impact of factor A is concerned, we may conclude that Algorithm 3 in Table 3 for the initialization of a feasible simplex, when compared with Algorithm 2 in Table 2: (i) has slightly increased the percentage of successful runs (with *E*_*e*_ = 0.010); (ii) has a small negative impact on *Y*_*e*_ (with *E*_*e*_ = −2.628), meaning that the number of function evaluations has slightly decreased; and (iii) also has a small negative impact on time (with *E*_*e*_ = −0.001), i.e., the time has been reduced.

Finally, factor D has little impact on the response variables. When using the Lévy distribution to generate the *n* worst vertices of the simplex, instead of the standard shrinkage strategy, the percentage of successful runs has slightly increased (with *E*_*e*_ = 0.010), the number of function evaluations and the time have also suffered small increases (*E*_*e*_ = 12.767 and *E*_*e*_ = 0.001 respectively).

The effects of interactions between factors are estimated using formula (13) as well. Relative to the variable *Y*_*p*_, the estimated (interaction) effects, *E*_*e*_, in absolute value, range from 0.005 to 0.014. This seems to show that the differences are considered normal variations just due to chance. There are some small values of interaction effect of AD, BC, BD and BCD but they are in general smaller than the effects of each factor separately. The same is true relative to the interaction effects on both *Y*_*e*_ and *Y*_*t*_.

### Normal variation

To better analyze the variation of the effects to check if they vary normally or if some of them are significantly kept away from the others, we carry out the plotting of the effects, using their absolute values and the half-normal probability plot. The horizontal axis of the half-normal plot displays the absolute value of the estimated effects and the vertical axis displays the cumulative probability of obtaining a value less or equal to a certain target. The values for the plotting are displayed in Table 7.

**Table 7:** Values for the half-normal probability plots.

	*Y*_*p*_		*Y*_*e*_		*Y*_*t*_		
	source	∣*E*_*e*_∣	source	∣*E*_*e*_∣	source	∣*E*_*e*_∣	cum. prob.^§^
1	AB	0.005	ABC	0.077	BC	0.0001	0.033
2	AC	0.005	AC	0.297	ABC	0.0002	0.100
3	ABC	0.006	ABCD	0.783	AC	0.0002	0.167
4	ABCD	0.006	ABD	1.731	ABCD	0.0003	0.233
5	ACD	0.007	A	2.628	BD	0.0003	0.300
6	CD	0.008	AB	3.237	AB	0.0004	0.367
7	ABD	0.008	BC	3.882	A	0.001	0.433
8	D	0.010	BD	4.526	ABD	0.001	0.500
9	A	0.010	ACD	6.234	AD	0.001	0.567
10	BCD	0.011	AD	7.003	ACD	0.001	0.633
11	AD	0.012	CD	9.637	D	0.001	0.700
12	BC	0.013	D	12.767	BCD	0.001	0.767
13	BD	0.014	BCD	14.185	CD	0.002	0.833
14	B	0.024	B	82.315	B	0.008	0.900
15	C	0.158	C	110.260	C	0.012	0.967

^§^ midpoints of 15 probability segments

Values for the half-normal probability plots

**Fig 2:**
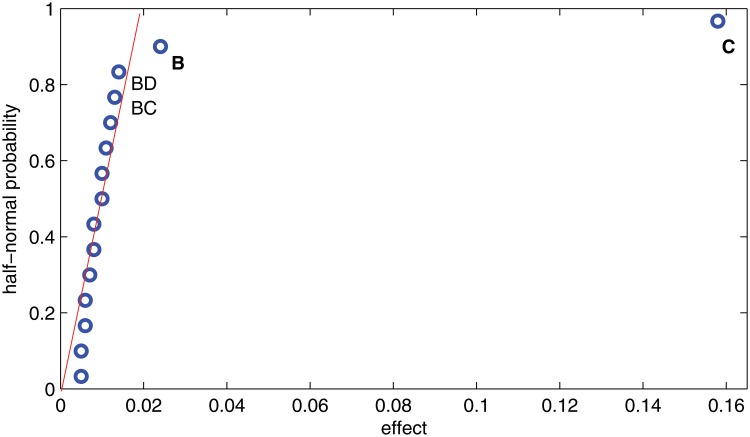
Half-normal plot of effects for *Y*_*p*_.

**Fig 3:**
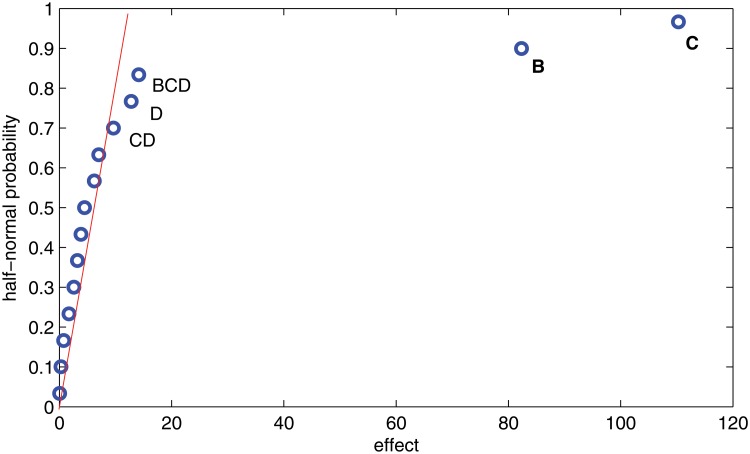
Half-normal plot of effects for *Y*_*e*_.

From the half-normal probability plot, we aim to identify the important effects, i.e., the factors that significantly affect the values of the response variables, in a statistical sense. Unimportant effects tend to have a normal distribution centered near zero while important effects tend to have a normal distribution centered at their large effect values. Fig 2 contains the half-normal probability plot of the effects for variable *Y*_*p*_. It is observed that factor B and in particular factor C fall off to the right of the straight line that emanates from the origin and fits the near zero values. Note that the majority of the effects/points are near zero and fall along the line meaning that their variations are due to normal causes, like noise, and are considered unimportant. The effects that are likely to be important fall out and are far from the line. Fig 3 displays the same type of plot of the effects for variable *Y*_*e*_. Again, factors B and C have big effects when compared to the others. To confirm these conclusions in a statistical sense, an analysis of variance is carried out in the next subsection.

**Fig 4:**
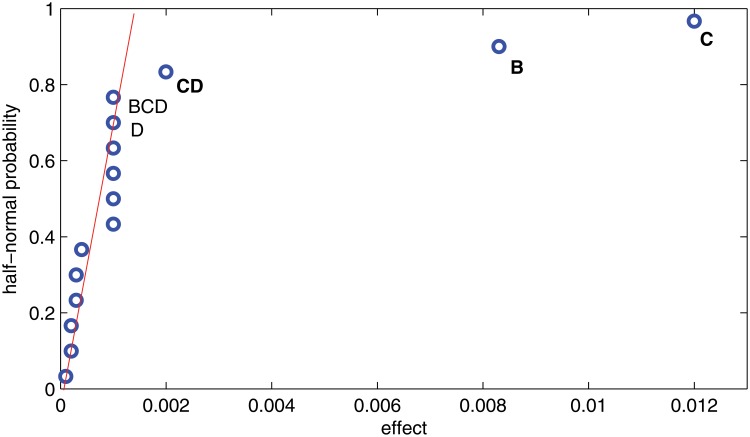
Half-normal plot of effects for *Y*_*t*_.

Fig 4 shows that factors B and C seem to be important, and it looks like that interaction CD could be important. This matter is investigated in the next subsection.

### Analysis of variance

The statistical analysis of DoE relies on the analysis of variance (ANOVA), a collection of statistical models that partition the observed variance on the response variable (variation relative to the mean value) into components according to the factors that the experiment is testing. The statistical analysis of the four factor effects, as well as their interaction effects, on the response variables is carried out using ANOVA. Statistical hypotheses tests are needed to determine if the factor/interaction effects are significant. Sums of squares (SoS)—sum of all the squared effects—for each factor/interaction must be computed, and are related to the estimated effects in (13), as follows:

$$\begin{array}{c} SoS=2^{N_{f}-2}{\left(E_{e}\right)}^{2}. \end{array}$$ (14)

For each response variable, each effect that is likely to be significant is tested with the following statistical hypotheses:

H_0_: The variable is not significantly affected by the variation in the factor/interaction, i.e., the effect produced on the variable is not statistically significant.
H_1_: The variable is significantly affected by the variation in the factor/interaction (the effect produced on the variable is statistically significant).

In ANOVA, the total variation relative to the mean value, designated by ‘Total SoS’, is partitioned into two components. One is due to the model and the other to the residual

$$\begin{array}{c} \text{Total}\;\text{SoS}={\text{SoS}}_{model}+{\text{SoS}}_{residual} \end{array}$$

where the effects that are likely to be significant (and are to be tested for statistical significance) are incorporated in the model and the remaining ones with near zero values are used to estimate the error, or residual. The two largest effects (B and C) are the ones that fall out the straight line in the half-normal plot for the response variable *Y*_*p*_ and since we also aim to check the effect of the interaction BC, the SoS components are:

$$\begin{array}{c} {\text{SoS}}_{model}={\text{SoS}}_{B}+{\text{SoS}}_{C}+{\text{SoS}}_{BC} \end{array}$$ (15)

$$\begin{array}{ccc} {\text{SoS}}_{residual} & = & {\text{SoS}}_{A}+{\text{SoS}}_{D}+{\text{SoS}}_{AB}\\ & & +\;\cdots +{\text{SoS}}_{BCD}+{\text{SoS}}_{ABCD}. \end{array}$$ (16)

The values for the ANOVA for *Y*_*p*_ are shown in Table 8. The SoS for the components of the model and for the residual are shown in the second column of the table. The third column contains de degrees of freedom (df) associated with SoS—one df for each factor/interaction since only two-levels are present –, the fourth column shows the mean square (MS) (the SoS divided by df) and the ratio of MS of an effect over the MS of the residual, giving the ‘statistic’ F value (Fval), is on the fifth column. Finally, the last column of the table shows the probability of getting an Fval as high as that computed, due to chance alone, known in the literature as ‘Prob. value > F’ (P. val). The Fval is compared to the reference F-distribution with the same df. We decided to use a 1% risk (the risk of erroneously rejecting the null hypothesis H_0_ when it is true), also known as level of significance, in these hypotheses tests. This means that if the computed ‘Fval’ exceeds the critical value of the reference F-distribution for the 1% risk, H_0_ should be rejected, and we are 99% confident that the ‘response variable’ is significantly affected by the effect of that factor/interaction, in the model. Based on the values of ‘P. val’ in Table 8, we conclude that only the ‘Fval’ of factor C exceeds the critical value 9.33 of *F*_1,12_ (1 df for the SoS in numerator and 12 df for the SoS in the denominator) that corresponds to a 1% risk. (It even exceeds the critical value of *F*_1,12_ for a 0.1% risk.) Thus, the two strategies identified as low and high levels of factor C are able to produce statistically significant differences on the percentage of successful runs.

**Table 8:** ANOVA for *Y*_*p*_.

source	SoS	df	MS	Fval	P. val
B	2.40E-03	1	2.40E-03	7.5	> 0.01^†^
C	1.00E-01	1	1.00E-01	312.7	< 0.001^§^
BC	6.78E-04	1	6.78E-04	2.1	> 0.01
residual	3.85E-03	12	3.21E-04		
Total SoS	1.07E-01	15			

^†^ critical point of *F*_1,12_ = 9.33

^§^ critical point of *F*_1,12_ = 18.64

ANOVA for *Y*_*p*_

Next, we show the ANOVA carried out for the response variable *Y*_*e*_. The SoS components for the model include factors B and C and to be able to check the importance of the effect due to the interaction BCD, we had to incorporate factor D as well in the analysis. For a more complete checking, interaction CD is also included,

$$\begin{array}{ccc} {\text{SoS}}_{model} & = & {\text{SoS}}_{B}+{\text{SoS}}_{C}+{\text{SoS}}_{D}\\ & & +\;{\text{SoS}}_{CD}+{\text{SoS}}_{BCD} \end{array}$$ (17)

$$\begin{array}{ccc} {\text{SoS}}_{residual} & = & {\text{SoS}}_{A}+{\text{SoS}}_{AB}\\ & & +\;\cdots +{\text{SoS}}_{ACD}+{\text{SoS}}_{ABCD}. \end{array}$$ (18)

The values for the ANOVA for *Y*_*e*_ are shown in Table 9. Based on the ‘P. val’ reported in the table, we may conclude that from the tested factors (B, C and D) and interactions (CD and BCD), only the interaction CD effect is not significant, since the associated ‘Fval’ does not exceed the critical value 10.04 of *F*_1,10_ (1 df for the SoS in numerator and 10 df for the SoS in the denominator) that corresponds to the 1% risk. This means that although the levels of the factors C and D affect variable *Y*_*e*_ significantly, the effect caused by one of the factors on *Y*_*e*_ does not depend on the level of the other. We note here that for both factors B and C we are 99.9% confident that the number of function evaluations to locate each root is significantly affected since their ‘Fval’ highly exceed the critical value of *F*_1,10_ for the 0.1% risk.

**Table 9:** ANOVA for *Y*_*e*_.

source	SoS	df	MS	Fval	P. val
B	2.71E+04	1	2.71E+04	468.8	< 0.001^†^
C	4.86E+04	1	4.86E+04	841.1	< 0.001
D	6.52E+02	1	6.52E+02	11.3	< 0.01^§^
CD	3.71E+02	1	3.71E+02	6.4	> 0.01
BCD	8.05E+02	1	8.05E+02	13.9	< 0.01
residual	5.78E+02	10	5.78E+01		
Total SoS	7.81E+04	15			

^†^ critical point of *F*_1,10_ = 21.04

^§^ critical point of *F*_1,10_ = 10.04

ANOVA for *Y*_*e*_

However, when the interaction BCD is considered, the effect of one of the factors B, C or D is significantly affected, in statistical sense, by the levels of the other two factors, as far as the average number of function evaluations is concerned. The ‘Fval’ associated with BCD exceeds the reference critical value as reported in Table 9. We may observe how factors B, C and D interact. We notice that when B and C are at low level, and D varies from low to high level, *Y*_*e*_ decreases from 208.6 (average of 209.2 and 207.9) to 202.1 (average of 201.0 and 203.1), while when B is at high level and C remains at low level, the variation of D from low to high level makes *Y*_*e*_ to increase from 285.1 (average of 286.6 and 283.6) to 297.9 (average of 302.7 and 293.1).

ANOVA for the response variable *Y*_*t*_ is the following. To be able to analyze the effect of CD we have to include factor D in the model. Interaction BCD is also integrated in the model. Thus, the components of the SoS for the model and SoS for the residual are:

$$\begin{array}{ccc} {\text{SoS}}_{model} & = & {\text{SoS}}_{B}+{\text{SoS}}_{C}+{\text{SoS}}_{D}\\ & & +\;{\text{SoS}}_{CD}+{\text{SoS}}_{BCD} \end{array}$$ (19)

$$\begin{array}{ccc} {\text{SoS}}_{residual} & = & {\text{SoS}}_{A}+{\text{SoS}}_{AB}+{\text{SoS}}_{AC}\\ & & +\;\cdots +{\text{SoS}}_{ACD}+{\text{SoS}}_{ABCD}. \end{array}$$ (20)

Table 10 contains the values of the ANOVA for *Y*_*t*_. Note that the computed values of ‘Fval’ for factor D, and interactions CD and BCD do not exceed the critical value 10.04 of *F*_1,10_ for a 1% risk. Thus, the associated H_0_ could not be rejected. Only the H_0_ associated with factors B and C, for *Y*_*t*_, should be rejected even at a 0.1% risk. The main conclusions are the following: (i) the two strategies related with the setting of the simplex *γ* parameters affect significantly the response variable (time to compute a root); and (ii) the two tested strategies related with solving the problem when the simplex collapses also affect significantly the time to locate each root.

**Table 10:** ANOVA for *Y*_*t*_.

source	SoS	df	MS	Fval	P. val
B	2.78E-04	1	2.78E-04	300.8	< 0.001^†^
C	5.60E-04	1	5.60E-04	606.3	< 0.001
D	5.10E-06	1	5.10E-06	5.5	> 0.01^§^
CD	8.66E-06	1	8.66E-06	9.4	> 0.01
BCD	7.16E-06	1	7.16E-06	7.8	> 0.01
residual	9.24E-06	10	9.24E-07		
Total SoS	8.68E-04	15			

^†^ critical point of *F*_1,10_ = 21.04

^§^ critical points of *F*_1,10_ = 10.04

ANOVA for *Y*_*t*_

## Comparison of results

This section aims to show some performance comparison between the proposed repulsion algorithm based on a Nelder-Mead type local search and some other available techniques for locating multiple roots of a system of nonlinear equations. First, we compare our results with those reported in [7]. The results are summarized in Table 11 that shows:

- ‘*SR*’ (%), the percentage of runs (out of 30) where all the roots were located;
- ‘*N*_r_’, the average number (out of 30) of located roots per run;
- ‘*NFE*_r_’, the average number of function evaluations required to locate a root;
- ‘*T*_r_’, the average time required to locate a root (in seconds);

relative to twelve problems of the previous set. From the proposed variants of the N-M algorithm, and according to the experimental testing of the previous section, we choose the one that combines the high levels of factors A, B, C, and D. We note that the results reported in [7] were obtained using a repulsion algorithm that uses the metaheuristic known as the harmony search as the local procedure. The differences between the three tested algorithms lie in the penalty term used to modify the merit function and to define the repulsion area as roots are computed. The penalty terms involve an inverse ‘erf’ function [7], an ‘exp’ function [8] and the ‘coth’ function [6]. These results are shown in the last three sets of four columns of Table 11. The first set of four columns of the table contains the results obtained by our study. We may conclude that our method notably wins in efficiency and is comparable to the others in terms of percentage of successful runs.

**Table 11:** Comparison with the results reported in [7].

Problem	this study (A_+1_B_+1_C_+1_D_+1_)				inverse ‘erf’ penalty^†^				‘exp’ penalty^‡^				‘coth’ penalty^§^			
	*SR*	*aN*_r_	*aNFE*_r_	*aT*_r_	*SR*	*aN*_r_	*aNFE*_r_	*aT*_r_	*SR*	*aN*_r_	*aNFE*_r_	*aT*_r_	*SR*	*aN*_r_	*aNFE*_r_	*aT*_r_
NonD2	100	2.0	293	0.030	100	2.0	748	0.057	100	2.0	753	0.055	100	2.0	867	0.070
Trans	97	3.0	227	0.018	90	2.9	3,474	0.269	97	3.0	3,266	0.231	90	2.9	10,627	0.833
Himmelblau	83	8.8	234	0.028	60	8.6	6,539	0.504	0	5.9	6,420	0.536	0	6.2	9,456	0.788
Geometry	80	1.8	320	0.029	53	1.4	6,291	0.482	43	1.4	6,332	0.452	63	1.6	10,273	0.811
Floudas	100	2.0	201	0.016	97	2.0	3,206	0.232	100	2.0	3,145	0.213	100	2.0	8,193	0.631
Merlet	70	12.6	209	0.034	100	13	967	0.081	20	11.4	902	0.137	10	10.1	1,146	0.111
Reactor	67	6.7	303	0.030	10	5.9	28,087	2.132	7	5.7	27,451	2.243	3	6.0	77,563	6.597
P1syst	100	2.0	344	0.027	100	2.0	2,181	0.183	97	2.0	1,139	0.080	100	2.0	6,151	0.476
Papersys	50	2.2	311	0.025	7	1.7	6,897	0.498	60	2.4	6,866	0.473	60	2.4	7,301	0.545
Casestudy5	100	2.0	470	0.059	83	2.1	3,780	0.295	100	2.0	3,471	0.252	23	3.1	4,393	0.363
Casestudy7	100	3.0	228	0.020	100	3.0	3,508	0.256	90	2.9	3,520	0.246	90	2.9	3,624	0.282
Manipulator	97	5.9	389	0.037	100	6.0	3,594	0.281	0	5.0	2,501	0.178	100	6.0	17,551	1.225

^†^ penalty function proposed in [7];

^‡^ penalty function proposed in [8];

^§^ penalty function proposed in [6]

*SR* is the percentage of runs that found all roots, *aN*_r_ is the average number of located roots per run, *aNFE*_r_ is the average number of function evaluations required to locate a root, and *aT*_r_ is the average time required to locate a root.

We aim to further compare the efficiency of our algorithm with the results reported in [3] and [8], regarding five problems of the previous set. In [8], the authors propose a biased random-key genetic algorithm to minimize the merit function coupled with a penalty approach to modify the merit function as roots are found. The penalty term is an ‘exp’ type function and is implemented with two parameters. One aims to create an area of repulsion around previously found roots and the other aims to penalize points inside the repulsion area. In [3], a multistart approach that uses a gradient-based BFGS variant as the local search procedure is implemented. The statistics reported by the authors correspond to three multistart implementations that differ in the stopping rule adopted by the algorithm. Here, we report only the second best value. The results are shown in Table 12. Beside the statistics used in the previous table, we also show the average number of N-M local search calls, *L*_cal_, for our algorithm. In the table, ‘–’ means that the data is not available in the referenced paper. In these comparisons we increase the parameters *k*_*R*max_ and the maximum of *k*_uns_ of the repulsion algorithm to 100 and 25, respectively, since the computational budget is not an important issue. Although our algorithm did not find all roots in all runs, when solving problems Reactor and Robot, it requires less function evaluations than the other two in comparison. While the average time needed to find each root is significantly lower than that of the heuristic proposed in [8], it exceeds the value of the average time reported in [3] (ranging from one to eight times), on problems Floudas, Merlet and Reactor (*R* = 0.960).

**Table 12:** Comparison with the results reported in [3] and [8].

Problem	this study (A_+1_B_+1_C_+1_D_+1_)				in [8]			in [3]		
	*aN*_r_	*aNFE*_r_	*aT*_r_	*aL*_cal_	*aN*_r_	*aNFE*_r_	*aT*_r_	*aN*_r_	*aNFE*_r_	*aT*_r_
Himmelblau	9.0	235	0.029	46	9.0	253,877	0.500	–	–	–
Floudas	2.0	233	0.019	48	2.0	211,652	0.304	2.0	2,137	0.015
Merlet	13.0	200	0.031	76	13.0	401,021	3.486	13.0	354	0.004
Reactor^†^	6.7	343	0.037	42	7.0	–	0.693	7.0	795	0.007
Reactor^‡^	2.6	275	0.023	51	3.0	933,515	12.853	3.0	3,156	0.027
Robot	15.7	1,219	0.275	64	16.0	–	63.516	–	–	–

^†^ *R* = 0.960;

^‡^ *R* = 0.945.

*aN*_r_ is the average number of located roots per run, *aNFE*_r_ is the average number of function evaluations required to locate a root, *aT*_r_ is the average time required to locate a root, and *aL*_cal_ represents the average number of N-M local search calls.

## Performance on standard benchmarks

Since our proposals for the N-M variant seem promising when compared with those of the classical N-M, we decided to extend them to solving more complex and real application systems [18, 20–22]. The benchmark set is a difficult set of problems. For these experiments, *k*_*R*max_ and the maximum of *k*_uns_ (to stop the Repulsion Algorithm) are set to 75 and 15 respectively, the parameters *ρ*_*ε*_ and *δ* in the definition of the penalty term in (4) are fixed at 0.01 and 100 respectively, and the N-M algorithm is allowed to run for a maximum of 1000*n* iterations. We compare our results with two deterministic global search techniques, a traditional interval method that uses range testing and branching, designated as ‘HRB’, and a branch and prune approach denoted by ‘Newton’ in [20]. We also use the results produced by a continuation method designated by ‘CONT’ in [20] and those of a multiobjective evolutionary algorithm approach presented in [18], for comparison. It is mentioned that the algorithm has produced a constant number (≤ 200) of nondominated solutions in each run. We now describe each benchmark and the obtained results with some detail.

**Economics modeling application.** This is an economic modeling problem that can be tested for a variety of dimensions *n*:

$$\begin{array}{c} \left\{\begin{array}{cc} f_{i}=\left(x_{i}+\sum_{j=1}^{n-i-1}x_{j}x_{j+i}\right)x_{n}-c_{i}=0, & i=1,\ldots ,n-1\\ f_{n}=\sum_{j=1}^{n-1}x_{j}+1=0 & \end{array}\right.. \end{array}$$

We test for *n* = 4,5,6,7,8,9 and compare with the results available in [20] considering the set Ω = [−100,100]^*n*^. The results produced by the ‘Newton’ algorithm correspond to interval widths smaller than 1.0E-08. See Table 13. Our algorithm is able to identify several roots. We note here that a located root *ξ* is considered to be different from any other computed root if they differ in norm more than *ε* = 0.05. We observe that as *n* increases, the number of located roots decreases. This means that the difficulty in converging to a root is higher when *n* is larger and this is related with the performance of the N-M search procedure that deteriorates as *n* increases. While the computational effort (function evaluations and time) in ‘Newton’ greatly increases with *n*, the number of function evaluations in our algorithm grows at most by a factor of two, if the number of local search calls is the same (see, for example, the instances with *n* = 4,5,6,7). However, the total time essentially depends on the time required by the N-M procedure to locate each root.

**Table 13:** Comparison results using the economic modeling application.

	A_+1_B_+1_C_+1_D_+1_						‘Newton’ in [20]		‘CONT’^†^
Instance	*aN*_r_	*aNFE*_r_	*aT*_r_	*aL*_cal_	*aNFE*_tot_	*aT*_tot_	*NFE*_tot_	*T*_tot_ ^‡^	*T*_tot_ ^§^
*n* = 4	71.5	806	2.65	75	90,414	548.1	3,513	0.21	≈ 1
*n* = 5	58.1	1,192	2.34	75	245,332	466.7	17,932	1.22	≈ 6
*n* = 6	48.4	1,942	2.68	75	424,651	579.3	129,819	8.20	≈ 50
*n* = 7	28.1	3,794	1.93	75	780,006	435.9	650,178	46.59	≈ 990
*n* = 8	8.0	5,363	0.97	57	843,728	165.2	4689,636	352.80	–
*n* = 9	0.2	3,374	0.53	17	318,780	47.5	39901,284	3,311.42	–

^†^ information available in [20];

^‡^ time on a Sun Sparc 10 workstation;

^§^ time on a DEC 5000/200

*NFE*_tot_ gives the total number of function evaluations, *T*_tot_ is the total time in seconds, *aNFE*_tot_ and *aT*_tot_ are the averaged values of the total number of function evaluations and total time in seconds, respectively (out of the 30 runs), and *aL*_cal_ represents the average number of N-M local search calls.

**Neurophysiology application.** This is an application from neurophysiology with *n* = 6 and is defined by:

$$\begin{array}{c} \left\{\begin{array}{c} f_{1}=x_{1}^{2}+x_{3}^{2}-1=0\\ f_{2}=x_{2}^{2}+x_{4}^{2}-1=0\\ f_{3}=x_{5}x_{3}^{3}+x_{6}x_{4}^{3}-c_{1}=0\\ f_{4}=x_{5}x_{1}^{3}+x_{6}x_{2}^{3}-c_{2}=0\\ f_{5}=x_{5}x_{1}x_{3}^{2}+x_{6}x_{2}x_{4}^{2}-c_{3}=0\\ f_{6}=x_{5}x_{3}x_{1}^{2}+x_{6}x_{4}x_{2}^{2}-c_{4}=0 \end{array}\right. \end{array}$$

where *c*_*i*_ can be chosen at random. We test this application with *c*_*i*_ = 0, *i* = 1, …, 4 and consider three intervals [−10,10]^6^, [−100,100]^6^ and [−1000,1000]^6^, as proposed in [20]. See Table 14. In [18] (with 300 initial points and after 200 generations), the multiobjective algorithm produces a set of about 200 nondominated solutions after 28.90 seconds. (The time reported in [18] is based on a 2.4-GHz Intel Duo Core CPU with 2-GB RAM.) The results show that the larger the interval the smaller is the number of located roots. Once again we observe that as the number of located roots increases the larger is the time required to converge to a root (see the *aT*_r_ values). This is an important characteristic of the Repulsion Algorithm since the minimization of the modified penalty merit function gets harder as the number of penalty terms increases.

**Table 14:** Comparison results using a neurophysiology application.

	A_+1_B_+1_C_+1_D_+1_						‘Newton’ in [20]		‘HRB’^†^	‘CONT’^†^
Interval	*aN*_r_	*aNFE*_r_	*aT*_r_	*aL*_cal_	*aNFE*_tot_	*aT*_tot_	*NFE*_tot_	*T*_tot_ ^‡^	*T*_tot_	*T*_tot_ ^§^
[−10, 10]^6^	55.1	2,370	4.33	75	357,417	621.2	15,116	0.91	28.84	5.02
[−10^2^, 10^2^]^6^	11.4	4,528	0.72	64	624,163	116.8	193,647	11.69	–	–
[−10^3^, 10^3^]^6^	0.2	5,760	0.51	15	186,658	17.7	2389,594	172.71	–	5.02

^†^ information available in [20];

^‡^ time on a Sun Sparc 10 workstation;

^§^ time on a DEC 5000/200

*NFE*_tot_ gives the total number of function evaluations, *T*_tot_ is the total time in seconds, *aNFE*_tot_ and *aT*_tot_ are the averaged values of the total number of function evaluations and total time in seconds, respectively (of the 30 runs), and *aL*_cal_ represents the average number of N-M local search calls.

**Interval arithmetic benchmarks.** The following two sets of functions are benchmarks from the interval arithmetic area. They are designated as instances i1 and i5 and are defined as

$$\begin{array}{c} \left\{\begin{array}{c} f_{1}=x_{1}-0.25428722-0.18324757x_{3}x_{4}x_{9}\\ f_{2}=x_{2}-0.37842197-0.16275449x_{1}x_{6}x_{10}\\ f_{3}=x_{3}-0.27162577-0.16955071x_{1}x_{2}x_{10}\\ f_{4}=x_{4}-0.19807914-0.15585316x_{1}x_{6}x_{7}\\ f_{5}=x_{5}-0.44166728-0.19950920x_{3}x_{6}x_{7}\\ f_{6}=x_{6}-0.14654113-0.18922793x_{5}x_{8}x_{10}\\ f_{7}=x_{7}-0.42937161-0.21180486x_{2}x_{5}x_{8}\\ f_{8}=x_{8}-0.07056438-0.17081208x_{1}x_{6}x_{7}\\ f_{9}=x_{9}-0.34504906-0.19612740x_{6}x_{8}x_{10}\\ f_{10}=x_{10}-0.42651102-0.21466544x_{1}x_{4}x_{8} \end{array}\right. \end{array}$$

and

$$\begin{array}{c} \left\{\begin{array}{c} f_{1}=x_{1}-0.25428722-0.18324757x_{3}^{3}x_{4}^{3}x_{9}^{3}+x_{3}^{4}x_{9}^{7}\\ f_{2}=x_{2}-0.37842197-0.16275449x_{1}^{3}x_{6}^{3}x_{10}^{3}+x_{6}^{7}x_{10}^{4}\\ f_{3}=x_{3}-0.27162577-0.16955071x_{1}^{3}x_{2}^{3}x_{10}^{3}+x_{2}^{4}x_{10}^{7}\\ f_{4}=x_{4}-0.19807914-0.15585316x_{1}^{3}x_{6}^{3}x_{7}^{3}+x_{1}^{4}x_{6}^{7}\\ f_{5}=x_{5}-0.44166728-0.19950920x_{3}^{3}x_{6}^{3}x_{7}^{3}+x_{3}^{7}x_{6}^{4}\\ f_{6}=x_{6}-0.14654113-0.18922793x_{5}^{3}x_{8}^{3}x_{10}^{3}+x_{5}^{4}x_{10}^{7}\\ f_{7}=x_{7}-0.42937161-0.21180486x_{2}^{3}x_{5}^{3}x_{8}^{3}+x_{5}^{4}x_{8}^{7}\\ f_{8}=x_{8}-0.07056438-0.17081208x_{1}^{3}x_{6}^{3}x_{7}^{3}+x_{6}^{7}x_{7}^{4}\\ f_{9}=x_{9}-0.34504906-0.19612740x_{6}^{3}x_{8}^{3}x_{10}^{3}+x_{6}^{4}x_{8}^{7}\\ f_{10}=x_{10}-0.42651102-0.21466544x_{1}^{3}x_{4}^{3}x_{8}^{3}+x_{1}^{7}x_{8}^{4} \end{array}\right. \end{array}$$

respectively [20]. The set Ω for instance i1 is [−2,2]^10^ and for i5 is [−1,1]^10^. Both have *n* = 10 and one root in Ω. Our results as well as those available in [20] are reported in Table 15. We note that in [18] a set of nondominated solutions are obtained after 300 generations and 39.08 seconds when solving the benchmark i1 (using an initial population of 500 points), and 366.40 seconds when solving i5. We are able to accelerate our iterative process by setting the maximum of *k*_uns_ to 5 since the root is computed during the first iterations of the Repulsion Algorithm. From the results shown in the Table 15 we may conclude that the number of function evaluations and time required to converge to a root are comparable to that of ‘Newton’. Since the repulsion algorithm’s paradigm requires restarting again and again, searching the search space and looking for other roots, the corresponding total values may become large, depending on the number of restarts. Nevertheless, those totals are comparable to the totals of ‘HRB’.

**Table 15:** Comparison results using interval arithmetic benchmarks.

		A_+1_B_+1_C_+1_D_+1_						‘Newton’ in [20]		‘HRB’^†^	
Instance		*aN*_r_	*aNFE*_r_	*aT*_r_	*aL*_cal_	*aNFE*_tot_	*aT*_tot_	*NFE*_tot_	*T*_tot_ ^‡^	*NFE*_tot_	*T*_tot_
i1	^(*a*)^	1	1,194	0.14	15	701,994	102.8	1,760	0.06	77,380	14.28
	^(*b*)^	1	1,237	0.15	5	242,597	18.5				
i5	^(*a*)^	1	1,167	0.15	15	284,258	31.8	1,132	0.08	154,948	33.58
	^(*b*)^	1	1,179	0.16	5	95,394	11.3				

^†^ information available in [20];

^‡^ time on a Sun Sparc 10 workstation

^(*a*)^ results produced with the maximum of *k*_uns_ set to 15; ^(*b*)^ results produced with the maximum of *k*_uns_ set to 5.

**Chemical equilibrium application.** This problem addresses the equilibrium of the products of a hydrocarbon combustion process which, reformulated in the variable space, is [21]:

$$\begin{array}{c} \left\{\begin{array}{c} f_{1}=x_{1}x_{2}+x_{1}-3x_{5}=0\\ f_{2}=2x_{1}x_{2}+x_{1}+x_{2}x_{3}^{2}+R_{5}x_{2}-R_{1}x_{5}+2R_{7}x_{2}^{2}+R_{4}x_{2}x_{3}+R_{6}x_{2}x_{4}=0\\ f_{3}=2x_{2}x_{3}^{2}+2R_{2}x_{3}^{2}-8x_{5}+R_{3}x_{3}+R_{4}x_{2}x_{3}=0\\ f_{4}=R_{6}x_{2}x_{4}+2x_{4}^{2}-4R_{1}x_{5}=0\\ f_{5}=x_{1}x_{2}+x_{1}+R_{7}x_{2}^{2}+x_{2}x_{3}^{2}+R_{5}x_{2}+R_{2}x_{3}^{2}+x_{4}^{2}-1+R_{3}x_{3}+R_{4}x_{2}x_{3}+R_{6}x_{2}x_{4}=0 \end{array}\right. \end{array}$$

where $R_{1}=10,R_{2}=0.193,R_{3}=0.002597/\sqrt{40},R_{4}=0.003448/\sqrt{40}$, *R*_5_ = 0.00001799/40, $R_{6}=0.0002155/\sqrt{40},R_{7}=0.00003846/40$. The problem has one root and we tested it for three different sets: [10^−4^,10^2^]^5^, [10^−4^,10^3^]^5^ and [10^−4^,10^8^]^5^. The statistics computed from the results of our algorithm are shown in Table 16, where we report the results available in [20, 21] as well. In [18], 500 generations and an initial population of 500 points are considered to obtain a set of about 200 nondominated solutions in 32.71 seconds. Since we look for just one root, we first set the maximum of *k*_uns_ to 10. Although the root was located in only 10% of the runs, when the set Ω = [10^−4^,10^8^]^5^ is used, our iterative process takes on average 2,482 function evaluations (and 0.19 seconds) to converge to the solution. While the branch and prune technique ‘Newton’ requires a total of 52,236 function evaluations (and 6.32 seconds) to converge to the required solution, our iterative process computes 179,951 function values when the limit of unsuccessful iterations is set to 10, but it computes 55,913 function values when the limit of unsuccessful iterations is 5.

**Table 16:** Comparison results using the chemical equilibrium application.

		A_+1_B_+1_C_+1_D_+1_						results in [21]		‘Newton’ in [20]	
Interval		*aN*_r_	*aNFE*_r_	*aT*_r_	*aL*_cal_	*aNFE*_tot_	*aT*_tot_	*Nit*_tot_	*T*_tot_ ^†^	*NFE*_tot_	*T*_tot_ ^‡^
[10^−4^, 10^2^]^5^	^(*a*)^	0.52	1,167	0.10	13	144,785	12.0	631	31.7	–	–
	^(*b*)^	0.1	1,090	0.10	5	56,093	4.6				
[10^−4^, 10^3^]^5^	^(*a*)^	0.13	2,031	0.16	10	160,096	11.5	–	–	–	–
	^(*b*)^	0.03	1,124	0.11	5	56,307	4.6				
[10^−4^, 10^8^]^5^	^(*a*)^	0.1	2,482	0.19	10	179,951	12.5	–	–	52,236	6.32
	^(*b*)^	0.07	1,424	0.13	5	55,913	4.6				

^†^ time on a HP-730 workstation;

^‡^ time on a Sun Sparc 10 workstation

*Nit*_tot_ is the total number of iterations; ^(*a*)^ results produced with the maximum of *k*_uns_ set to 10; ^(*b*)^ results produced with the maximum of *k*_uns_ set to 5.

We now use the following five examples together with the ‘Neurophysiology application’ (with Ω = [−10,10]^6^) and ‘Interval arithmetic’ benchmark i1 (with Ω = [−10,10]^10^) to analyze and confirm the performance behavior of our choices for the N-M variant, with a set of problems different from the one used during the DoE analysis.

**Bratu system.** This is a polynomial system about *x*_1_, …, *x*_*n*_ that arises from the discretization (with a mesh size of *h*) of the differential equation model for nonlinear diffusion phenomena taking place in combustion and semiconductors—a two-point boundary value problem—[3, 23]:

$$\begin{array}{c} \left\{\begin{array}{cc} f_{i}=x_{i-1}-2x_{i}+x_{i+1}+h^{2}e^{x_{i}}=0, & i=1,\ldots ,n \end{array}\right. \end{array}$$

where *x*_0_ = *x*_*n*+1_ = 0 and $h=\frac{1}{n+1}$. In the set [0, 5]^*n*^, there are two roots for all *n*. We tested this problem with *n* = 5.

**Brown almost-linear system.** This is a system with *n* variables

$$\begin{array}{c} \left\{\begin{array}{cc} f_{i}=x_{i}+\sum_{j=1}^{n}x_{j}-\left(n+1\right)=0, & i=1,\ldots ,n-1\\ f_{n}=\prod_{j=1}^{n}x_{j}-1=0 & \end{array}\right. \end{array}$$

which has two real roots if *n* is even and three roots if *n* is odd. It is a ill-conditioned and difficult to solve for some standard methods [21, 22]. In the interval [−1,1]^*n*^ there is one root when *n* = 10.

**Broyden tridiagonal system.** This is a sparse polynomial system that has at least two real roots [24],

$$\begin{array}{c} \left\{\begin{array}{cc} f_{i}=\left(3-2x_{i}\right)x_{i}-x_{i-1}-2x_{i+1}+1=0, & i=1,\ldots ,n \end{array}\right. \end{array}$$

where *x*_0_ = *x*_*n*+1_ = 0. We set *n* = 5 and Ω = [−1,2]^5^.

**Resistive circuit problem** This system of *n* nonlinear equations

$$\begin{array}{c} \left\{\begin{array}{cc} f_{i}=2.5x_{i}^{3}-10.5x_{i}^{2}+11.8x_{i}+\sum_{j=1}^{n}x_{j}-i=0, & i=1,\ldots ,n \end{array}\right. \end{array}$$

describes a nonlinear resistive circuit containing *n* tunnel diodes [25]. The set Ω is defined by [−10,10]^*n*^ and we test this circuit problem with *n* = 5 and *n* = 7.

**Yamamura problem.** Another polynomial system tested for *n* = 10 [3, 23, 25]

$$\begin{array}{c} \left\{\begin{array}{cc} f_{i}=x_{i}-\frac{1}{2n}\left(\sum_{j=1}^{n}x_{j}^{3}+i\right)=0 & i=1,\ldots ,n \end{array}\right. \end{array}$$

which has three roots in the interval [−5,5]^10^.

All the experiments were run 30 times with each problem and the reported results are average values over the 30 runs. First, we aim to show that the herein proposed parameter values, defined in (8), make the N-M algorithm to work better. Overall, the number of located roots has in general increased and the number of function evaluations and time have not increased, except when solving problem Brown. See the first two sets of three columns (identified with A_+1_B_+_C_+1_D_+1_ and A_+1_B_−1_C_+1_D_+1_) in Table 17. We note that the problems now used are in general of larger dimension than those used during the DoE analysis. The last set of three columns of the table shows the results obtained when the shrinking strategy is selected, A_+1_B_−1_C_+1_D_−1_ (as opposed to the strategy defined in (12)). Improvements are obtained only on the problems Brown and Broyden, when compared to A_+1_B_+_C_+1_D_+1_. Here, we consider an improvement when the number of located roots has increased, or the number of function evaluations has decreased provided that a tie in the number of roots is reported.

**Table 17:** Comparing simplex N-M parameters.

	A_+1_B_+1_C_+1_D_+1_			A_+1_B_−1_C_+1_D_+1_			A_+1_B_−1_C_+1_D_−1_		
Problem	*aN*_r_	*aNFE*_r_	*aT*_r_	*aN*_r_	*aNFE*_r_	*aT*_r_	*aN*_r_	*aNFE*_r_	*aT*_r_
Neurophys.	30.7	2,181	1.345	28.9	2,239	1.111	30.1	2,007	1.120
Interval	1.0	1,611	0.199	1.0	2,125	0.220	1.0	4,167	0.368
Bratu	1.8	2,618	0.203	1.7	2,634	0.202	1.7	1,825	0.141
Brown	1.0	109	0.013	1.0	94	0.011	1.0	105	0.014
Broyden	1.5	1,355	0.120	1.4	1,083	0.097	1.6	747	0.068
Circuit_5	3.5	1,786	0.175	3.0	2,079	0.189	2.5	767	0.072
Circuit_7	1.6	1,643	0.174	0.8	2,160	0.217	0.6	2,232	0.219
Yamamura	3.0	1,910	0.232	3.0	2,195	0.258	3.0	2,093	0.247

A_+1_B_+1_C_+1_D_+1_ uses the values defined in (8) and A_+1_B_−1_C_+1_D_+1_ (as well as A_+1_B_−1_C_+1_D_−1_) use the standard values in (7).

Second, we show that the strategy defined in (12), in the context of our proposals, is able to locate in general more roots than the classic shrinking methodology, using now this new set of problems. These two variants are identified by A_+1_B_+_C_+1_D_+1_ and A_+1_B_+_C_+1_D_−1_ respectively in the Table 18. We also include the results of another variant that uses a uniformly distributed random number instead of a number drawn from the Lévy distribution. We observe that the variant A_+1_B_+_C_+1_D_+1_ wins on four problems. It is able to locate more roots than the other two in comparison on problems Broyden, Circuit_5 and Circuit_7 and is more efficient than the other two on the problem Interval (for the same number of located roots). The variant A_+1_B_+_C_+1_D_−1_ wins on problem Neurophysiology and is more efficient on the problems Brown and Yamamura. The variant A_+1_B_+_C_+1_D_+1(*Uniform*)_ wins only on the problem Bratu.

**Table 18:** Comparing Lévy and Uniform distributions with classical shrinkage.

	A_+1_B_+1_C_+1_D_+1_			A_+1_B_+1_C_+1_D_+1(*Uniform*)_			A_+1_B_+1_C_+1_D_−1_		
Problem	*aN*_r_	*aNFE*_r_	*aT*_r_	*aN*_r_	*aNFE*_r_	*aT*_r_	*aN*_r_	*aNFE*_r_	*aT*_r_
Neurophys.	30.7	2,181	1.345	30.6	1,996	1.092	30.9	2,086	1.197
Interval	1.0	1,611	0.199	1.0	2,596	0.256	1.0	3,471	0.323
Bratu	1.8	2,847	0.222	2.0	2,521	0.192	1.9	2,978	0.241
Brown	1.0	109	0.013	1.0	115	0.013	1.0	108	0.014
Broyden	1.5	1,355	0.120	1.1	463	0.043	1.3	947	0.088
Circuit_5	3.5	1,786	0.175	2.1	736	0.069	2.6	1,259	0.129
Circuit_7	1.6	1,643	0.174	0.9	1,511	0.150	0.7	1,430	0.146
Yamamura	3.0	1,900	0.230	3.0	1,806	0.225	3.0	1,755	0.219

D_+1(*Uniform*)_ means that in Eq (12) the Lévy distribution term $L(\alpha )\sigma_{j}^{i}$ is replaced by a multiple of a random number uniformly distributed in [−1, 1], i.e., by 0.1 *U*([−1, 1]).

The results reported in Table 19 are used to show that the variant A_+1_B_+_C_+1_D_+1_ that uses the golden ratio Φ to define the step size in order to generate points around the best vertex of the simplex (see (11)) outperforms the other two variants A_+1_B_+_C_+1(*random*)_D_+1_ and A_+1_B_+_C_−1_D_+1_ in comparison. C_+1(*random*)_ means that in Eq (11) the step size is a multiple of a random number uniformly distributed in [0, 1], and C_−1_ represents the strategy that breaks the N-M iterative process when the simplex has collapsed.

**Table 19:** Comparing step sizes for random point generation and ‘break’ strategy.

	A_+1_B_+1_C_+1_D_+1_			A_+1_B_+1_C_+1(*random*)_D_+1_			A_+1_B_+1_C_−1_D_+1_		
Problem	*aN*_r_	*aNFE*_r_	*aT*_r_	*aN*_r_	*aNFE*_r_	*aT*_r_	*aN*_r_	*aNFE*_r_	*aT*_r_
Neurophys.	30.7	2,181	1.345	30.4	2,072	1.241	2.9	561	0.060
Interval	1.0	1,611	0.199	1.0	1,346	0.157	1.0	1,213	0.145
Bratu	1.8	2,847	0.222	2.0	597	0.059	1.2	486	0.046
Brown	1.0	109	0.013	1.0	621	0.047	0.0	n.a.	n.a.
Broyden	1.5	1,355	0.120	1.1	427	0.041	1.0	423	0.041
Circuit_5	3.5	1,786	0.175	2.9	737	0.072	2.2	688	0.067
Circuit_7	1.6	1,643	0.174	0.5	1,449	0.155	0.3	1,286	0.128
Yamamura	3.0	1,900	0.230	3.0	2,311	0.263	1.8	1,845	0.220

C_+1(*random*)_ means that in Eq (11) the step size is a multiple of a random number uniformly distributed in [0, 1] (i.e., 0.01 *U*([0, 1])); n.a. means not applicable.

Finally, we aim to show that the maximum target value, *ρ*_*ε*_, for the radius of the repulsion area *ρ* (see the definition in (4) and in Subsection ‘Parameters Setting’) slightly affects the performance of the algorithm. In this section and until now we have set the fixed value *ρ*_*ε*_ = 0.01 when solving the eight problems. We test two other different values: 0.1 and 0.001. Table 20 contains the results produced by the variant A_+1_B_+_C_+1_D_+1_. We may conclude that the values 0.01 and 0.1 give better results than 0.001. Between the two best values, 50% of the results are better with *ρ*_*ε*_ = 0.01 and the remaining are better with *ρ*_*ε*_ = 0.1.

**Table 20:** Effect of *ρ*_*ϵ*_ on the Repulsion Algorithm.

	*ρ*_*ϵ*_ = 0.001			*ρ*_*ϵ*_ = 0.01			*ρ*_*ϵ*_ = 0.1		
Problem	*aN*_r_	*aNFE*_r_	*aT*_r_	*aN*_r_	*aNFE*_r_	*aT*_r_	*aN*_r_	*aNFE*_r_	*aT*_r_
Neurophys.	29.3	2,277	1.350	30.7	2,181	1.345	32.1	2,038	5.067
Interval	1.0	2,131	0.234	1.0	1,611	0.199	1.0	1,210	0.150
Bratu	1.8	2,618	0.203	1.8	2,847	0.222	1.9	2,892	0.246
Brown	1.0	119	0.019	1.0	109	0.013	1.0	99	0.014
Broyden	1.3	1,761	0.180	1.5	1,355	0.120	1.5	1,774	0.155
Circuit_5	3.4	2,152	0.273	3.5	1,786	0.175	3.8	1,727	0.167
Circuit_7	1.2	1,731	0.201	1.6	1,643	0.174	0.9	1,534	0.158
Yamamura	3.0	1,910	0.232	3.0	1,900	0.230	3.0	2,523	0.312

Effect of *ρ*_*ϵ*_ on the Repulsion Algorithm, using the N-M variant A_+1_B_+1_C_+1_D_+1_.

## Conclusions

A repulsion algorithm is presented for locating multiple roots of a system of nonlinear equations. The proposed algorithm relies on a penalty merit function that depends on two parameters. One aims to scale the penalty and the other adjusts the radius of the repulsion area, so that convergence to previously located minimizers of the merit function is avoided. For each randomly generated point in the search space, the algorithm invokes the N-M algorithm to exploit the region for a new minimizer. In the N-M local search context, several alternative strategies have been incorporated in the algorithm aiming to enhance the quality of the solutions and improve efficiency. To analyze the effect of these strategies on the overall performance of the repulsion algorithm, measured by three criteria—the percentage of successful runs, the number of function evaluations and the time required to compute each root -, a two-level factorial design of experiments is carried out. Four factors at two levels each are manipulated and tested to analyze their statistical significance. During these computational experiments, a set of sixteen benchmark problems is used. The values of the response variables used in the factorial design of experiments correspond to the averaged values after 30 independent runs produced by solving the sixteen problems.

From the statistical analysis, we may conclude with 99% confidence that:

- the number of function evaluations to locate each root and the time to locate each root have significantly increased when the simplex parameters are changed from the classical values to those based on the golden ratio and dimension;
- the percentage of successful runs, the number of function evaluations to locate each root and the time to locate each root have significantly been affected when the two approaches to overcome the simplex degeneracy are tested, in particular, they have increased when the new strategy of generating *n* vertices around the best vertex with probability 0.75 is used, instead of just ‘breaking’ the cycle;
- the number of function evaluations to locate each root has significantly increased when the Lévy distribution is used to generate *n* vertices around the best vertex instead of using the classical shrinking procedure;
- the number of function evaluations to locate each root has significantly decreased by the interaction that occurs when the simplex parameters change from the classical values to those based on the golden ratio and *n*, when the generation of *n* vertices around the best vertex with probability 0.75 is used, instead of the ‘break’ strategy, and when the Lévy distribution is used to generate *n* vertices around the best one as opposed to the shrinking strategy.

We have also tested our method with other more complex and realistic problems. The results show that our method is capable of converging to the multiple solutions of those problems with a moderate computational effort. To undergo a variety of other modifications to the N-M algorithm using a limited number of experimental units, a fractional factorial experimental design as opposed to the herein operated full factorial design will be used. The techniques for investigating the variation in experiments brought by the Taguchi methods will be explored.
